# Disparities in Smoking Cessation Treatment Among Immigrants and Potential Barriers to Care

**DOI:** 10.1007/s40615-025-02443-4

**Published:** 2025-04-22

**Authors:** Raymond Hsu, Sebastian T. Tong

**Affiliations:** 1 University of Washington School of Medicine, Seattle, USA; 2 University of Washington Department of Family Medicine, Seattle, USA

**Keywords:** Immigrants, Smoking cessation, Tobacco

## Abstract

**Introduction:**

Despite lower smoking prevalence than U.S.-born individuals, immigrants face disparities in smoking cessation treatment. Insurance status and limited English proficiency (LEP) impede their healthcare access, but their impact on tobacco cessation treatment remains unclear. This study examines smoking cessation treatment differences between immigrants and U.S.-born individuals and explores potential barriers to access for immigrants.

**Methods:**

Data from the 2018 and 2020 Medical Expenditure Panel Survey were analyzed. Variables regarding demographics, insurance status, time spent in the U.S., and English proficiency were examined. Chi-squared and binomial logistic regression were used to compare smoking cessation treatment between U.S.-born individuals and immigrants and to understand differences within the immigrant subsample.

**Results:**

Among 3727 daily smokers surveyed, 247 were immigrants (6.6%). Immigrants had lower odds than U.S.-born individuals of being asked about tobacco use by healthcare providers (OR = 0.66, 95% CI [0.47–0.93]). Within the immigrant subgroup, those without insurance had lower odds of being asked about tobacco use (OR = 0.26, 95% CI [0.11–0.62]) and had lower odds of being advised to quit smoking in the past year (OR = 0.29, 95% CI [0.12–0.69]). Immigrants who had LEP were less likely to have ever been asked about tobacco use (OR = 0.44, 95% CI [0.20–0.94]).

**Conclusion:**

These findings highlight that persistent disparities in healthcare, namely insurance status and language barriers, may hinder access to smoking cessation treatment services in immigrant populations. Future research should focus on developing interventions that address these barriers while promoting cultural competency and linguistically appropriate care.

## Introduction

Foreign-born immigrants to the United States account for approximately 14% of the country’s entire population, according to data published by the Pew Research Center in 2024. The overall prevalence of smoking among immigrants is less than U.S.-born individuals, consistent with the “healthy immigrant effect” [1, 2]. However, the issue of smoking among immigrants must not be overlooked, especially given the heterogeneity of immigrant populations. For example, immigrants from Russia, the Middle East, Asia, and North Africa are significantly more likely to smoke than U.S.-born individuals [3]. Asian immigrants who spend 10 years or more in the U.S. are much more likely to smoke than those who only spend less than 10 years living in the U.S. [4]. This heterogeneity stems from factors such as country of origin, education level, and gender norms [3]. Smoking among a variety of immigrant subgroups is linked to high rates of cardiovascular disease [5] and non-small cell lung cancer [6].

Immigrants to the U.S. also face a variety of challenges that prevent their ability to access quality healthcare. Despite the implementation of the Affordable Care Act (ACA), many immigrants, particularly those who are undocumented or have limited English proficiency (LEP), remain at risk of being uninsured [7, 8]. Consequently, being uninsured among immigrants has led to an underutilization of primary care and preventative health [9, 10]. For smokers, this is concerning because smoking cessation benefits from longitudinal care [11]. Furthermore, LEP can impede communication between patients and healthcare providers, which may interfere with treatment adherence, limit access to preventative care and screening services, and lead to poor chronic disease management [12]. Ultimately, language barriers pose a significant risk for poorer mental and physical health outcomes among immigrants [13].

Little is known about how these barriers to accessing healthcare may affect immigrants’ abilities to access treatment for their tobacco cessation. Much of the current literature surrounding tobacco cessation treatment among immigrants highlights culturally sensitive, text-based messaging strategies and innovations for tobacco cessation among immigrants [14, 15]. Although novel alternatives are being developed, little is known about how widely recognized smoking cessation services differ in accessibility between immigrants and U.S.-born individuals, as well as the barriers contributing to these differences. One study conducted at two New York City hospitals in 2018 demonstrated that immigrants are less likely to be prescribed nicotine replacement therapy compared to non-immigrants [16]. Although this study investigates nicotine replacement therapy, one of the most widely established pharmacotherapies, many other strategies are available. Additionally, the study was conducted at two hospitals in New York City, limiting its generalizability to other clinical settings, particularly primary care clinics.

The objective of this study is to characterize health disparities among immigrants nationwide regarding smoking cessation treatment. The first part of this study compares U.S.-born daily smokers with foreign-born daily smokers to assess the receipt of specific smoking cessation methods. The second part examines a sample of foreign-born daily smokers to determine how potential barriers to care such as insurance status, English proficiency, and duration of residence in the U.S. influence their access to smoking cessation interventions.

## Methods

A dataset was created using data from the Medical Expenditure Panel Survey (MEPS), a publicly available, nationally representative, longitudinal survey that administers large-scale questionnaires to the U.S. civilian noninstitutionalized population regarding demographics, healthcare utilization, and health conditions. Overlapping panels of household members are interviewed five times over 2 calendar years, and pertinent data is compiled into a dataset for each calendar year. MEPS is particularly valuable for its oversampling of minorities including Blacks, Hispanics, and Asian Americans [17]. More information about the methodology can be found in Cohen et al.’s work [18]. This study utilized and combined MEPS data from the 2018 Full-Year Consolidated Data File and the 2020 Full-Year Consolidated Data File, the most recent datasets containing our variables of interest. Relevant variables concerning demographic history, immigration history, language proficiency, insurance status, and smoking cessation treatment history were extracted into one dataset.

All participants who were at least 18 years of age and smoked every day for the past year of taking the survey were included. Furthermore, survey respondents who did answered all the questions of interest were included in the final sample. Among the 58,266 respondents to either the 2018 or 2020 Full-Year Consolidated Data Files, 3727 were included in the final dataset.

Due to limited respondents in certain answer choices, specific variables were re-coded. Education level was condensed to indicate whether the respondent received a high school diploma or equivalent. The number of years the respondent had resided in the U.S. was condensed to indicate whether they had been in the U.S. for 15 years or more. Finally, a new English proficiency variable was established: immigrants who exclusively spoke English or answered, “Very well” or “Well” to the English proficiency question were categorized as “English Proficient,” while those who answered “Not well” or “Not at all” were classified as “LEP” for Limited English Proficiency.

In the first part of the study, the primary independent variable was whether the respondent was born in the United States. The dependent variables included whether a doctor or other healthcare professional had ever asked patients about their tobacco or smoking use, advised them to quit smoking in the past year, recommended quitting smoking using medications in the past year, or suggested alternative methods such as a telephone helpline or group counseling in the past year. Demographic comparisons between these two groups were made using chi-squared tests, followed by comparisons of each dependent variable. Finally, binary logistic regression was conducted to adjust for demographic factors and to produce odds ratios with a 95% confidence interval, indicating the likelihood of the dependent variable outcomes based whether they were born in the U.S.

The second part of the study included the subset of respondents from the first part that were born outside the U.S. The independent variables examined were whether they were insured for the entire year, had been in the U.S. for 15 or more years, and were proficient in English. The dependent variables remained the same as in the first part. Chi-squared tests were used to compare demographics between the groups for each independent variable. Subsequent comparisons between each dependent and independent variable were also made using chi-squared tests. Lastly, binary logistic regression was performed to examine the association between insurance status, years in the US, and limited English proficiency with the likelihood of the dependent variable’s occurrence. The model adjusted for age, sex, race, and education level as covariates, producing odds ratios with 95% confidence intervals.

Analyses were conducted using IBM SPSS Statistics, Version 29.0.1.0. The Westat Institutional Review Board reviewed and approved MEPS (MPA M- 1531). Since this study utilized anonymous secondary data, no additional ethical review was required.

## Results

Our sample consisted of 3727 participants. The mean age of the sample was 50.1 (SD = 15.2). 54.7% were male, 78.0% were white, and 82.2% received a high school diploma, G.E.D., or a higher qualification. The total sample of those not born in the U.S. consisted of 247 immigrants, comprising 6.6% of the overall sample. Among the immigrant subsample, the average age was 53.0 (SD = 12.5). 69.2% were male, 66.8% were white, and 69.6% of the population received at least a high school diploma. Furthermore, 15.4% were uninsured for all of 2018 or 2020, 21.9% of the immigrant population had lived in the United States for less than 15 years, and 21.5% had LEP.

Chi-squared analysis of the demographics examined the relationship between immigrant status and variables such as age, sex, race, and education (table not shown). We found that, compared to U.S.-born individuals, immigrants were more likely to be older (*p* < 0.001), male (*p* < 0.001), Asian (*p* < 0.001), and less educated (*p* < 0.001).

Chi-squared analyses of the outcomes in Table 1 found statistically significant differences between immigrants and non-immigrants in the U.S. Compared to U.S.-born individuals, immigrants to the U.S. were less likely to have ever been asked about their tobacco or smoking use (*p* < 0.001). Within the immigrant subsample, immigrants who are uninsured were less likely to have ever been asked about their tobacco or smoking use (*p* < 0.001), less likely to have been advised to quit in the past year (*p* < 0.001), and less likely to have been advised to use medications for smoking cessation in the past year (*p* = 0.045) compared to immigrants with insurance. Immigrants who lived in the United States for less than 15 years were less likely to have been advised to quit smoking in the past year (*p* = 0.036) compared to immigrants who lived in the United States for 15 years or more. Immigrants who had LEP were less likely to be asked about their tobacco or smoking use (*p* = 0.010) and less likely to be advised to use medications for smoking cessation in the past year (*p* = 0.018) compared to those proficient in English. Other outcomes can be found in Table 1.

**Table 1:** Descriptive analysis of sample by smoking cessation strategy

	Ever asked about tobacco			Advised to quit			Advised with meds			Advised with other method		
	Yes, *n* (%)	No, *n* (%)	p^*^	Yes, *n* (%)	No, *n* (%)	p^*^	Yes, *n* (%)	No, *n* (%)	p^*^	Yes, *n* (%)	No, *n* (%)	p^*^
Full sample (*n* = 3727)												
Immigrant												
Yes	183 (74.1)	64 (25.9)	** < 0.001**	145 (58.7)	102 (41.3)	0.986	80 (32.4)	167 (67.6)	0.179	61 (24.7)	186 (75.3)	0.79
No	2884 (82.9)	596 (17.1)		2041 (58.6)	1439 (41.4)		988 (28.4)	2492 (71.6)		886 (25.5)	2594 (74.5)	
Immigrants only (*n* = 247)												
Uninsured												
Yes	17 (44.7)	21 (55.3)	** < 0.001**	10 (26.3)	28 (73.7)	** < 0.001**	7 (18.4)	31 (81.6)	**0.05**	5 (13.2)	33 (86.8)	0.07
No	166 (79.4)	43 (20.6)		135 (64.6)	74 (35.4)		73 (34.9)	136 (65.1)		56 (26.8)	153 (73.2)	
Years in the US												
< 15 years	36 (66.7)	18 (33.3)	0.159	25 (46.3)	29 (53.7)	**0.04**	13 (24.1)	41 (75.9)	0.14	8 (14.8)	46 (85.2)	0.06
15 + years	147 (76.2)	46 (23.8)		120 (62.2)	73 (37.8)		67 (34.7)	126 (65.3)		53 (27.5)	140 (72.5)	
English proficiency												
LEP	32 (60.4)	21 (39.6)	**0.010**	26 (49.1)	27 (50.9)	0.107	10 (18.9)	43 (81.1)	**0.02**	8 (15.1)	45 (84.9)	0.07
Proficient	151 (77.8)	43 (22.2)		119 (61.3)	75 (38.7)		70 (36.1)	124 (63.9)		53 (27.3)	141 (72.7)	

Values in bold indicate statistically significant data

^*^Chi-squared *p*-value

The binomial logistic regression analysis in Table 2 demonstrates that, even when controlling for demographic variables such as age, sex, race, and education, immigrants had lower odds than U.S.-born individuals to have ever been asked about tobacco or smoking use (OR = 0.66, 95% CI [0.47–0.93]). Within the immigrant subgroup, immigrants who were uninsured had lower odds than immigrants who were insured to have ever been asked about tobacco or smoking use (OR = 0.26, 95% CI [0.11–0.62]) and had lower odds of being advised to quit smoking in the past year (OR = 0.29, 95% CI [0.12–0.69]). Immigrants who had LEP had lower odds to have ever been asked about tobacco or smoking history (OR = 0.44, 95% CI [0.20–0.94]). Other findings from the binomial logistic regression analysis can be found in Table 2.

**Table 2:** Association between smoking cessation strategy and barriers to accessing care, adusting for demographic variables

	Ever asked about tobacco		Advised to quit		Advised with meds		Advised with other method	
	OR (95% CI)	p^*^	OR (95% CI)	p^*^	OR (95% CI)	p^*^	OR (95% CI)	p^*^
Full sample (*n* = 3727)								
Immigrant								
Yes	**0.66 (0.47–0.93)**	**0.02**	0.93 (0.69–1.25)	0.636	1.24 (0.91–1.69)	0.174	1.00 (0.72–1.39)	0.984
No	Ref		Ref		Ref		Ref	
Immigrants only (*n* = 247)								
Uninsured								
Yes	**0.26 (0.11–0.62)**	**0**	**0.29 (0.12–0.69)**	**0.01**	0.57 (0.21–1.55)	0.271	0.50 (0.16–1.58)	0.239
No	Ref		Ref		Ref		Ref	
Years in the US								
< 15 years	1.15 (0.51–2.58)	0.733	0.95 (0.46–1.99)	0.889	0.69 (0.32–1.51)	0.353	0.55 (0.22–1.37)	0.198
15 + years	Ref		Ref		Ref		Ref	
English proficiency								
LEP	**0.44 (0.20–0.94)**	**0.03**	0.63 (0.31–1.29)	0.209	0.47 (0.21–1.06)	0.068	0.51 (0.16–1.58)	0.138
Proficient	Ref		Ref		Ref		Ref	

Values in bold indicate statistically significant data

^*^Binary Logistic Regression *p*-value also correcting for age, sex, race, and education

## Discussion

This study found that even when controlling for demographic variables, immigrants were less likely to be asked about tobacco use, a critical first step in smoking cessation interventions. Within the immigrant subgroup, those who were uninsured and those with limited English proficiency faced even greater barriers, particularly as it pertains to being asked about tobacco use. These findings align with existing literature identifying insurance status and language proficiency as key determinants of healthcare access and quality [10, 12]. Moreover, after controlling for demographic variables, we found no statistically significant differences in advice to quit using medications between immigrants and U.S.-born individuals. Historically, immigrants to the U.S. have faced discrimination and fear of authorities which limits the ability and willingness for immigrants to seek care, particularly among Asian Americans, Latin Americans, and undocumented immigrants [19, 20]. This study identifies further barriers which may preclude their abilities to seek tobacco cessation care.

It is imperative that efforts to improve tobacco cessation outcomes in our country prioritize care for immigrants, especially those who are uninsured and have limited English proficiency. Healthcare settings can enhance cultural competency among professionals [21] and linguistically appropriate services such as interpreters. Public health initiatives and policies should focus on the distinct needs of recent immigrants to expand insurance coverage, address social determinants of health, and facilitate better integration into our healthcare system.

This study has several limitations. Its cross-sectional design limits the ability to establish causation between immigrant groups and disparities in smoking cessation treatment. The reliance on self-reported survey data introduces the risk of recall bias, particularly regarding smoking and access to cessation resources. Furthermore, this study’s small sample may not fully represent the nation’s immigrant population, with documented low research participation rates among immigrant groups, especially Asian Americans [22] and the exclusion of undocumented immigrants from MEPS sampling. Additionally, it is unclear whether the MEPS questionnaire includes U.S. territories and dependencies as part of the United States. A larger study population of immigrants may reveal even more pronounced differences between immigrants and U.S.-born individuals. This study also did not comprehensively account for other possible cofounders including country of origin, state/city of residence, cultural beliefs, or social support as these variables are not available in MEPS [17].

Despite these limitations, this exploratory study provides valuable initial insights into smoking cessation among immigrants and highlights potential avenues for future research. These findings suggest the need for future research to deepen understanding of disparities in tobacco cessation treatment among immigrants. To establish stronger causal relationships, longitudinal studies could be conducted. It would also be valuable to explore whether interactions between variables, such as insurance status and English proficiency, jointly affect the likelihood of receiving smoking-related interventions. Future studies should aim to include more diverse immigrant samples to capture the broad socioeconomic and demographic heterogeneity within the U.S. immigrant population, or focus on specific immigrant subpopulations to explore the nuances within these groups. Addressing these facets of immigrant health is crucial since these varied experiences may influence quality and access to care.

In addition to the insights we can glean from the robust literature surrounding smoking cessation among racial and ethnic minorities, further research is necessary to investigate interventions addressing these disparities specifically among immigrants. Detailed exploration of challenges faced by different immigrant subgroups can inform more targeted, culturally adapted cessation interventions. Moreover, there is a need for research on community-based interventions involving immigrant communities to develop culturally tailored strategies that address unique community needs [23]. Effective culturally tailored interventions should reduce tobacco consumption in their target populations and be well-received to ensure cultural sensitivity. Examples of successful interventions include counseling focused on Korean family values [24] and utilizing bilingual Latina health educators tailored to Latino values and culture [25]. These interventions may complement or potentially replace well-established tobacco cessation strategies explored in this study. Embracing these opportunities will not only enhance immigrant health equity but also strengthen public health across the nation.

## Data Availability

All data in this manuscript were from the Medical Expenditure Panel Survey, which is publicly available at https://meps.ahrq.gov.

## References

[1] Bosdriesz JR, Lichthart N, Witvliet MI, Busschers WB, Stronks K, Kunst AE. Smoking prevalence among migrants in the us compared to the us-born and the population in countries of origin. PLoS One. 2013;8(3):e58654. 10.1371/journal.pone.0058654. 23520525 10.1371/journal.pone.0058654 PMC3592805

[2] Acevedo-Garcia D, Pan J, Jun H-J, Osypuk TL, Emmons KM. The effect of immigrant generation on smoking. Soc Sci Med. 2005;61(6):1223–42. 10.1016/j.socscimed.2005.01.027. 15970233 10.1016/j.socscimed.2005.01.027

[3] Pampel F, Khlat M, Bricard D, Legleye S. Smoking among immigrant groups in the united states: prevalence, education gradients, and male-to-female ratios. Nicotine Tob Res. 2020;22(4):532–8. 10.1093/ntr/ntz022. 30759255 10.1093/ntr/ntz022

[4] Ra CK, Pehlivan N, Kim H, Sussman S, Unger JB, Businelle MS. Smoking prevalence among Asian Americans: associations with education, acculturation, and gender. Prev Med Rep. 2022;30:102035. 10.1016/j.pmedr.2022.102035. 36531113 10.1016/j.pmedr.2022.102035 PMC9747624

[5] Bolijn R, et al. The contribution of smoking to differences in cardiovascular disease incidence between men and women across six ethnic groups in Amsterdam, the Netherlands: the HELIUS study. Prev Med Rep. 2023;31:102105. 10.1016/j.pmedr.2022.102105. 36820382 10.1016/j.pmedr.2022.102105 PMC9938300

[6] Raz DJ, et al. Epidemiology of non-small cell lung cancer in Asian Americans: incidence patterns among six subgroups by nativity. J Thorac Oncol. 2008;3(12):1391–7. 10.1097/JTO.0b013e31818ddff7. 19057262 10.1097/JTO.0b013e31818ddff7 PMC5737739

[7] Porteny T, Ponce N, Sommers BD. Immigrants and the affordable care act: changes in coverage and access to care by documentation status. J Immigr Minor Health. 2022;24(1):86–94. 10.1007/s10903-020-01124-0. 33237344 10.1007/s10903-020-01124-0

[8] FoilesSifuentes AM, Robledo Cornejo M, Li NC, Castaneda-Avila MA, Tjia J, Lapane KL. The role of limited english proficiency and access to health insurance and health care in the affordable care act era. Health Equity. 2020;4(1):509–17. 10.1089/heq.2020.0057. 33376934 10.1089/heq.2020.0057 PMC7757700

[9] Cacari Stone L, Steimel L, Vasquez-Guzman E, Kaufman A. The potential conflict between policy and ethics in caring for undocumented immigrants at academic health centers. Acad Med. 2014;89(4):536–9. 10.1097/ACM.0000000000000187. 24556759 10.1097/ACM.0000000000000187 PMC6522141

[10] Saluja S, et al. Barriers to primary care after the affordable care act: a qualitative study of Los Angeles safety-net patients’ experiences. Health Equity. 2019;3(1):423–30. 10.1089/heq.2019.0056. 31448352 10.1089/heq.2019.0056 PMC6707030

[11] Burns RJ, Rothman AJ, Fu SS, Lindgren B, Vock DM, Joseph AM. Longitudinal care improves cessation in smokers who do not initially respond to treatment by increasing cessation self-efficacy, satisfaction, and readiness to quit: a mediated moderation analysis. Ann Behav Med. 2016;50(1):58–69. 10.1007/s12160-015-9732-1. 26373657 10.1007/s12160-015-9732-1 PMC4744132

[12] Pandey M, et al. Impacts of English language proficiency on healthcare access, use, and outcomes among immigrants: a qualitative study. BMC Health Serv Res. 2021;21(1):741. 10.1186/s12913-021-06750-4. 34311712 10.1186/s12913-021-06750-4 PMC8314461

[13] Jang Y, Yoon H, Park NS, Chiriboga DA. Health vulnerability of immigrants with limited english proficiency: a study of older Korean Americans. J Am Geriatr Soc. 2016;64(7):1498–502. 10.1111/jgs.14199. 27305524 10.1111/jgs.14199 PMC5441559

[14] Jiang N, et al. Development of a WeChat-based mobile messaging smoking cessation intervention for chinese immigrant smokers: qualitative interview study. JMIR Form Res. 2022;6(6):e36091. 10.2196/36091. 35771603 10.2196/36091 PMC9284363

[15] Pratt R, et al. Text message support for smoking cessation during Ramadan: a focus group study with somali immigrant Muslim men. Nicotine Tob Res. 2020;22(9):1636–9. 10.1093/ntr/ntz187. 31563964 10.1093/ntr/ntz187

[16] Chen J, Grossman E, Link A, Wang B, Sherman S. Disparities in hospital smoking cessation treatment by immigrant status. J Ethn Subst Abuse. 2020;19(1):44–57. 10.1080/15332640.2018.1446377. 29727588 10.1080/15332640.2018.1446377 PMC6215736

[17] Wang J, et al. Medical expenditure panel survey: a valuable database for studying racial and ethnic disparities in prescription drug use. Res Social Adm Pharm. 2008;4(3):206–17. 10.1016/j.sapharm.2007.06.018. 18794032 10.1016/j.sapharm.2007.06.018

[18] Cohen JW, Cohen SB, Banthin JS. The medical expenditure panel survey: a national information resource to support healthcare cost research and inform policy and practice. Med Care. 2009;47(7 Suppl 1):S44-50. 10.1097/MLR.0b013e3181a23e3a. 19536015 10.1097/MLR.0b013e3181a23e3a

[19] Woofter R, Sudhinaraset M. Differences in barriers to healthcare and discrimination in healthcare settings among undocumented immigrants by deferred action for childhood arrivals (DACA) status. J Immigr Minor Health. 2022;24(4):937–44. 10.1007/s10903-022-01346-4. 35226220 10.1007/s10903-022-01346-4 PMC9256563

[20] Young M-EDT, Tafolla S, Saadi A, Sudhinaraset M, Chen L, Pourat N. Beyond ‘chilling effects’: Latinx and Asian immigrants’ experiences with enforcement and barriers to health care. Med Care. 2023;61(5):306–13. 10.1097/MLR.0000000000001839. 36939228 10.1097/MLR.0000000000001839 PMC10079615

[21] Powers CA, Zapka J, Biello KB, O’Donnell J, Prout M, Geller A. Cultural competency and tobacco control training in US medical schools: many but missed opportunities. J Canc Educ. 2010;25(3):290–6. 10.1007/s13187-010-0090-1. 10.1007/s13187-010-0090-1 20559787

[22] Katigbak C, Foley M, Robert L, Hutchinson MK. Experiences and lessons learned in using community-based participatory research to recruit Asian American immigrant research participants. J Nurs Scholarsh. 2016;48(2):210–8. 10.1111/jnu.12194. 26836035 10.1111/jnu.12194 PMC5296612

[23] Leinberger-Jabari A, Golob MM, Lindson N, Hartmann-Boyce J. Effectiveness of culturally tailoring smoking cessation interventions for reducing or quitting combustible tobacco: a systematic review and meta-analyses. Addiction. 2024;119(4):629–48. 10.1111/add.16400. 38105395 10.1111/add.16400

[24] Kim SS, Kim S-H, Fang H, Kwon S, Shelley D, Ziedonis D. A culturally adapted smoking cessation intervention for Korean Americans: a mediating effect of perceived family norm toward quitting. J Immigr Minor Health. 2015;17(4):1120–9. 10.1007/s10903-014-0045-4. 24878686 10.1007/s10903-014-0045-4 PMC4250475

[25] Borrelli B, McQuaid EL, Novak SP, Hammond SK, Becker B. Motivating Latino caregivers of children with asthma to quit smoking: a randomized trial. J Consult Clin Psychol. 2010;78(1):34–43. 10.1037/a0016932. 20099948 10.1037/a0016932

